# Supplementary material for: An in-depth dataset of northwestern European arthropod life histories and ecological traits
Source: Biodivers Data J. 2025 Mar 11;13:e146785. doi: 10.3897/BDJ.13.e146785 (PMC11920819; doi:10.3897/BDJ.13.e146785)
Supplement: Supplementary material 2 — Linear regressions between temperature estimates [file bdj-13-e146785-s002.pdf]

S.2: linear regressions between temperature estimates

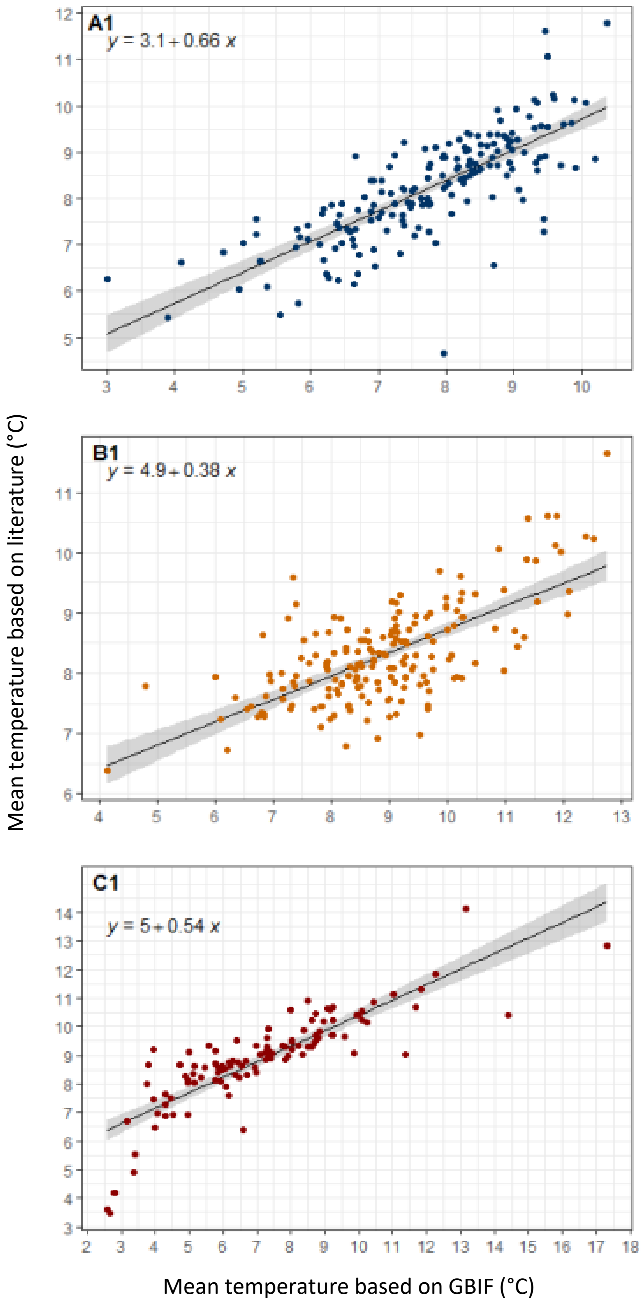

Figure S.2: Linear regressions between temperature estimates from literature and our calculations based on GBIF distribution data. Three different arthropod groups were assessed based on available literature data: (A) Carabidae, (B) Araneae and (C) Rhopalocera. Literature values were derived from Schweiger et al. (2014) and Bowler et al. (2017). The figure is adapted from the bachelor thesis of Uche Osajie (2021).
